# Supplementary material for: Contrasting responses to aridity by different-sized decomposers cause similar decomposition rates across a precipitation gradient
Source: eLife. 2024 Oct 15;13:RP93656. doi: 10.7554/eLife.93656 (PMC11479586; doi:10.7554/eLife.93656)
Supplement: Supplementary file 2. [file elife-93656-supp2.docx]

**Supplementary file 2**
Dissimilarity matrix between macro-decomposer assemblages of the different site-season combinations. Cells are filled on a green-yellow-orange-red scale with increasing Bray-Curtis dissimilarity.
